# Characterization of prenatally detected small Supernumerary Marker Chromosomes (sSMC) by molecular cytogenetic technique: FISH

**DOI:** 10.1186/1755-8166-7-S1-P55

**Published:** 2014-01-21

**Authors:** Bhumi Patel, Thomas Liehr, Manisha Desai, Bindu Parikh, Jayesh Sheth, Frenny Sheth

**Affiliations:** 1 FRIGE's Institute of Human Genetics, FRIGE House, Satellite, Ahmedabad-380 015, India; 2 Jena University Hospital, Institute of Human Genetics, Kollegiengasse 10, D-07743 Jena, Germany; 3 Satyam Hospital, 6/65 Nilam Park, Bapunagar, Ahmedabad India

## Background

Microscopically recognized chromosomal trisomies and monosomies are clinically well described. However, clinical effects in the unborn baby having imbalances of small chromosomal regions resulting from karyotypes containing small supernumerary marker chromosomes (SMCs) in addition to normal chromosomal count are less predictable. Moreover, due to extreme heterogeneity of sSMCs in size, structure and chromosomal origin, full characterization of sSMCs by molecular techniques – FISH has become imperative.

## Materials and methods

Two out of 1600 cases investigated showed sSMC during amniotic fluid (AF) analysis. In case-1, single sSMC (47,XN,+mar1)[100%] was detected during third gravida in a young couple having previous child with Down syndrome and second pregnancy ending in first trimester miscarriage. In case-2, two sSMCs were detected in the foetus of an elderly couple during primi gravida (i.e. 48,XN,+mar1,+mar2)[100%]. In both cases, fetal anomaly scan and triple marker study were normal. Parental chromosomal analysis at 500 band resolution was apparently normal at the time of prenatal study confirming *de novo* origin of the SMCs. Various FISH probes were applied such as (acro-)cenM-FISH, SRY, and subtel X/Ypter; besides immunohistochemistry using antiboies CENPB (all centromeres apart from Y) and CENPC (all active centromere) was done. Microdissection and reverse painting FISH was also performed for complete characterization.

## Results

Microdissection and reverse FISH was carried out in case-1 and showed signal only on the SMC. cenM-FISH did not yield any further information. Since the fetus had two X chromosomes, reverse FISH was carried out on a normal male control which gave signal on the #Yp. Additional probes specific to the SRY and subtel X/Y pter gave two signals confirming a neocentric inv dup(Y) i.e. 47,XN,+mar.ish inv dup(Y)(pter/Yp11.2::Yp11.2/pter)(SRY++)(subtelX/Y++)[100%]. The sSMC thus consisted of euchromatin exclusively. In case-2, sSMC were characterized as inv dup(13 or 21)(q10) and del(13 or 21)(q10) i.e. 48,XN,+inv dup(13 or 21)(q10)(CENPC++)(D13/21Z1+),+del(13 or 21)(q10)(CENPC+)(D13/21Z1+)[100%]. Marker chromosomes in case 2 solely consisted of heterochromatic material according to FISH.

## Conclusions

This shows that molecular technique FISH is one of the most powerful tools for precise identification and detail characterizations of SMCs and thereby providing valuable information to the families regarding genotype-phenotype correlation during prenatal diagnosis.

